# Periodontal Health of Pre-Dialysis Chronic Kidney Disease Patients in a Northeast Peninsular Malaysia Tertiary Hospital

**DOI:** 10.21315/mjms2020.27.1.11

**Published:** 2020-02-27

**Authors:** Hanim Afzan Ibrahim, Nur Karyatee Kassim, Fatimah Zahra Jamsari, Siti Lailatul Akmar Zainuddin, Muhammad Hafiz Hanafi, Azreen Syazril Adnan

**Affiliations:** 1 School of Dental Sciences, Universiti Sains Malaysia, Kelantan, Malaysia; 2 Chemical Pathology Department, Hospital Universiti Sains Malaysia, Kelantan, Malaysia; 3 School of Medical Sciences, Universiti Sains Malaysia, Kelantan, Malaysia; 4 Chronic Kidney Disease Resource Center, School of Medical Sciences, Universiti Sains Malaysia, Kelantan, Malaysia; 5 Management Science University (MSU) Medical Centre, Shah Alam, Selangor

**Keywords:** chronic kidney disease, chronic periodontitis, periodontal health, pre-dialysis

## Abstract

**Introduction:**

Chronic kidney disease (CKD) is associated with periodontal disease due to its hyperinflammatory state. Limited studies have explored the prevalence of periodontal disease among CKD patients in Malaysia.

**Objective:**

To assess the periodontal status of pre-dialysis CKD patients in Hospital Universiti Sains Malaysia.

**Methods:**

A total of 46 pre-dialysis CKD patients who attended the nephrology clinic at Hospital Universiti Sains Malaysia were enrolled in this study. Periodontal examination was performed using the periodontal probing depth (PPD), clinical attachment loss (CAL) and plaque index.

**Results:**

The majority of the CKD patients were Malay (95.7%) and 80.4% were males. The mean age of the patients was 58.5 years. Using PPD measurement, 37 (74.0%) of the patients had mild periodontitis, 9 (20.0%) had moderate periodontitis and 3 (6.0%) had no periodontitis. Based on CAL measurement, 12 (26%) patients had mild periodontitis, 29 (63.0%) had moderate periodontitis and 5 (11%) had severe periodontitis. The mean (standard deviation [SD]) value of mild and moderate-to-severe periodontitis by PPD measurement were 4.26 (0.26) and 5.24 (0.36), respectively. The mean of mild and moderate-to-severe periodontitis by CAL measurement were 2.66 (0.62) and 4.98 (0.73), respectively. There was no correlation between the periodontal parameters and estimated glomerular filtration rate (PPD: *r* = −0.160, *P* = 0.914; CAL: *r* = −0.135, *P* = 0.372; plaque index: *r* = 0.005, *P* = 0.974).

**Conclusion:**

This study revealed a greater prevalence and severity of chronic periodontitis among CKD patients. Thus, the periodontal health of CKD patients’ needs to be screened and monitored.

## Introduction

Chronic kidney disease (CKD) is defined as kidney damage from either pathological abnormalities/markers of damage or an estimated glomerular filtration rate (eGFR) < 60 mL/min/1.73 m^2^ for more than three months (1). CKD can be classified into five stages using guidelines of the Kidney Disease Outcomes Quality Initiative, the thresholds of eGFR within the CKD range and/or evidence of structural renal changes. The common causes of CKD are hypertension, diabetes mellitus, chronic glomerulonephritis, obstructive neuropathy, autoimmune disease and obesity (2).

The clinical signs and symptoms of CKD are dependent on the stage of the disease; they affect most of the body systems and are collectively called uraemia (3). CKD patients are known to be in a state of uraemia due to the limitation or reduction in the ability of the kidney to filter properly, which subsequently causes an increase in the level of toxic substances in the bloodstream. This is accompanied by an altered immune system because of impaired T- and B-lymphocytes, as well as monocytes and macrophages (4), resulting in reduced host response to sub-gingival gram-negative microbial aggression. Uraemia might also be associated with increased prevalence and severity of gingival inflammation and periodontitis, with increased dialysis vintage (4).

Uraemic patients have been found to have more dental problems in the oral mucosa, teeth, salivary glands (5) and jawbones than healthy controls, which seem to develop before dialysis. Xerostomia, uraemic stomatitis, periodontal disease and, maxillary and mandibular radiographic alterations can be observed in patients with CKD (6, 7). Periodontal disease is highly prevalent among patients with chronic renal failure (gingivitis to be specific) and uraemic patients with excessive plaque formation and poor oral hygiene (6, 8).

Periodontitis is defined as an infectious disease resulting in inflammation of the supporting tissues of the teeth, progressive attachment loss and bone loss (9) characterised by a pocket formation around the teeth and/or gum recession. According to Marcenes et al. (10), severe periodontitis affects up to 11% of the adult population globally, and about half of their study participants (48.5%) from the general population of Malaysia suffered from moderate-to-severe periodontitis.

Recently, periodontal disease has emerged as a nontraditional risk factor for CKD, as it can be a source of inflammatory products in systemic disease (11). Periodontitis has been associated with systemic diseases, including CKD, through the mechanism of systemic inflammation (12). In response to periodontal pathogens, the systemic mechanism spreads bacteria, antigens, endotoxins and inflammatory cytokines through the circulatory system, leading to endothelial dysfunction (13).

Moreover, the factors pre-disposing to periodontal disease and accelerating its progression are widespread in CKD. They encompass hyposalivation and xerostomia, impaired immunity and wound healing, alveolar bone destruction due to renal osteodystrophy, bleeding diathesis, diabetes mellitus, malnutrition and a state of general disability that impairs oral hygiene (14, 15).

Several studies showing evidence of the increased prevalence of periodontal disease in patients with CKD, focusing exclusively on end-stage renal disease patients on maintenance haemodialysis, have been published (8, 16). However, information available in Malaysia regarding the prevalence of periodontal disease in pre-dialysis CKD is limited. Moreover, periodontal status among CKD patients has not been monitored sufficiently in our setting. Hence, this study aimed to assess the periodontal status and periodontal parameters of pre-dialysis patients in Hospital Universiti Sains Malaysia (HUSM). In addition, this study aimed to investigate the correlation between periodontal parameters and the renal function status (i.e., eGFR) of study subjects.

## Methods

A cross-sectional study was conducted among pre-dialysis patients attending nephrology follow-up in the Chronic Kidney Disease Resource Centre and the Nephrology Specialist Clinic HUSM, Kubang Kerian, Kelantan, Malaysia between July 2018 and September 2018.

The inclusion criteria were patients aged 18 years and above who have at least 14 teeth, were diagnosed with CKD stage III (GFR of 30 mL/min/1.73 m^2^–59 mL/min/1.73 m^2^) or stage IV (GFR of 15 mL/min/1.73 m^2^–29 mL/min/1.73 m^2^) and have controlled diabetes mellitus (HbA1c < 6.5).

The exclusion criteria included the following: patients having any systemic diseases that acutely affect eGFR (rapid progressing glomerulonephritis, active glomerular disease, pregnancy, etc.) and/or oral health status (immunodeficiency syndrome, recurrent or active cancer); patients on medication that affect oral health status, such as immunosuppressive drugs (corticosteroid drugs or chemotherapy); and pregnant and lactating women. After the application of the inclusion and exclusion criteria, 46 patients were recruited for this study.

The sample size was calculated using the single proportion formula. In the calculation, the proportion was put as 0.09 based on the percentage of the pre-dialysis patient having severe periodontitis (2). The precision was set at 0.05, giving a sample size of 126. Purposive sampling method was done based on the list obtained from the e-Nephro database system.

Prior to the data collection process, the respondents received explanations regarding the purpose of the study and informed consent was obtained from the eligible respondents. The list of patients with CKD stage III or IV was obtained from the e-Nephro database system. eGFR was calculated using the CKD Epidemiology Collaboration equation as shown below:

$$\begin{array}{l} \text{eGFR}=141\times \text{min }{\left({\text{S}}_{\text{Cr}}/\kappa ,1\right)}^{\alpha }\times \text{max}{\left({\text{S}}_{\text{Cr}}/\kappa ,1\right)}^{-1.209}\\ \times 0.993^{\text{Age}}\times 1.018\;[\text{if female}]\times 1.159\;[\text{if Black}] \end{array}$$

κ = 0.7 (females) or 0.9 (males)
α = −0.329 (females) or −0.411 (males)
min = indicates the minimum of S_Cr_/κ or 1
max = indicates the maximum of S_Cr_/κ or 1

A proforma was used to collect the patients’ demographic details such as age, gender, ethnicity and other comorbidities. All the patients were identified by a code to ensure that only the researchers had access to their information. The examiner had been calibrated for periodontal assessment by a senior specialist and the inter- and the intra-examiner result showed that approximately 90% of the recording reproduced within ± 1.0 mm.

The following periodontal parameters were analysed:

1. Periodontal probing depth (PPD) was measured from the free gingival margin to the base of the periodontal pocket and on six sites per tooth (mesio-buccal, mid-buccal, disto-lingual, mesio-lingual, mid-lingual and disto-lingual sites).
2. Clinical attachment loss (CAL) was measured from a fixed point on a tooth from the cementoenamel junction (CEJ) to the gingival margin (GM) and adding the probing depth (PD) measurement: CAL = PPD + (CEJ GM).
3. Plaque index was used to record oral hygiene status by passing a periodontal probe over the cervical third to test the presence of plaque at four surfaces (buccal, mesial, distal and lingual) of the teeth.

Next, individuals were categorised according to the severity of their periodontitis based on established clinical criteria by the American Academy of Periodontology as mild, moderate and severe periodontitis (9).

Each individual was treated as needed according to the diagnosis established after the periodontal treatment. The treatment was performed in the Dental Clinic HUSM.

Data were entered and analysed with the Statistical Package for Social Science version 20.0. Descriptive statistics, such as mean, standard deviation (SD), frequency and percentages were calculated. Pearson’s correlation test was used to determine the correlation between each of periodontal parameter and eGFR in CKD patients.

## Results

**Figure 1:**
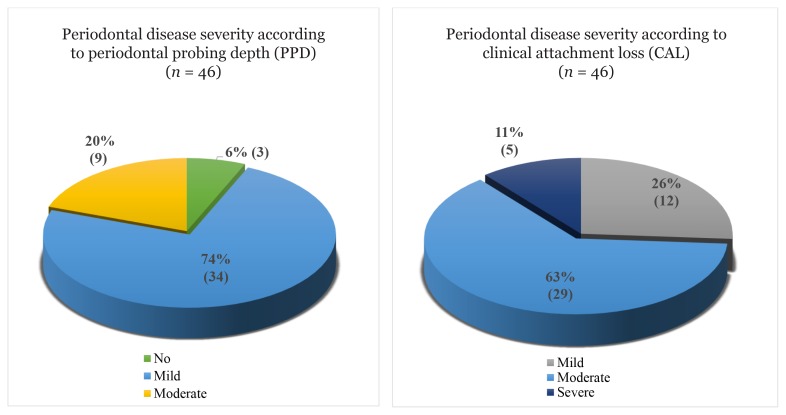
Periodontal disease severity according to PPD and CAL

**Figure 2:**
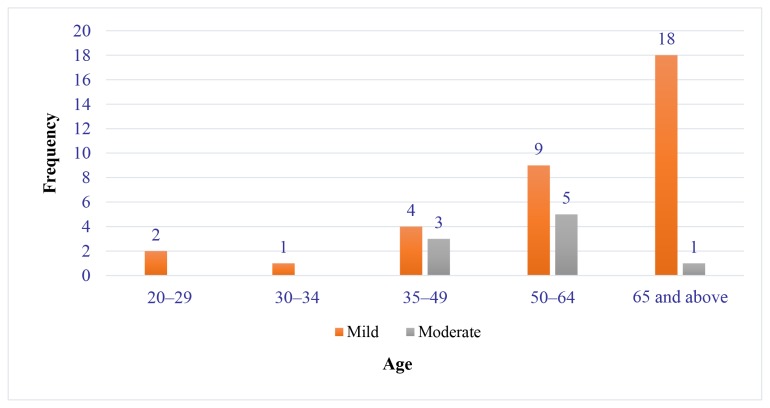
Distribution of severity of periodontitis based on periodontal probing depth according to age group

Among the 46 pre-dialysis patients, the majority were Malays (95.7%) and 37 (80.4%) were males. The mean age of the patients was 58.5 years. The patients in CKD stage III were 19, while 27 were in CKD stage IV. Table 1 demonstrates the sociodemographic characteristics of pre-dialysis CKD patients. The main comorbidities were hypertension and diabetes mellitus (Table 1). Evaluation of periodontal disease severity is shown in Figure 1. Most of the pre-dialysis patients (74%) had mild periodontitis based on PPD measurement (PPD = 4 mm), whereas 29 (63%) of them had moderate periodontitis based on CAL measurement (CAL = 3 mm–4 mm). Figure 2 shows that periodontitis (in term of PPD) is highly distributed in patients aged 35 years old and above with a greater number having mild periodontitis. Table 2 shows the SD of the periodontal parameter in pre-dialysis patients. The SD values of all cases of periodontitis with regard to PPD and CAL were 4.35 (0.99) and 3.97 (0.97), respectively. Overall, the mean plaque index of pre-dialysis patients was 47.9%, which indicated fair oral hygiene. However, we found no correlation between each of the periodontal parameter (PPD, CAL and plaque index) and eGFR measurement (Table 3).

## Discussion

Periodontitis has been shown to be prevalent and severe in CKD patients in Saudia Arabia (17), Poland (8) and India population (16) and has proved to be particularly advanced in end-stage renal disease patients that have undergone haemodialysis in respective studies. These studies revealed that the number of cases of periodontitis was higher in CKD population than the general healthy population. CKD has been shown to affect not only the general health of the patient but also oral and periodontal health. The periodontal health of the patient is assessed through the measurement of PPD and CAL.

The natural history of chronic periodontal disease can be viewed in stages, slowly progressing from its earliest form (gingivitis) to its severe form, characterised by irreversible pathological changes (periodontitis). During the ‘gingivitis’ stage, the disease is reversible if good oral hygiene is restored. However, if good oral hygiene is not restored, further destruction of the tissues can lead to periodontitis (18). Periodontitis is differentiated from gingivitis by the progressive breakdown of periodontal ligament fibres (loss of attachment) resulting in increased PDs and the resorption of alveolar bone, and the tissue damage that occurs is irreversible (19).

Most of the participants were Malay, as it is the pre-dominant race in Kelantan. CKD patients in the present study had the highest comorbidities of hypertension and diabetes mellitus (type II), as they are the most common causes of CKD through the mechanism of diabetic nephropathy and hypertensive nephrosclerosis (20).

PPD and CAL were evaluated for all the maxillary and mandibular teeth at six sites (buccal; mesial, mid, distal and lingual; mesial, mid-lingual) by graduated William’s probe. The results showed that most of the patients had moderate periodontitis (according to CAL) and mild periodontitis (according to PPD). Our finding is consistent with a study done in Saudi Arabia that found that 70% of pre-dialysis patients have moderate-to-severe periodontitis using CAL measurement (17). It has been established that CKD has a significant effect on the prevalence and severity of periodontitis. Moreover, factors that predispose and speed up the progression of periodontal disease are widespread in CKD. These factors include hyposalivation, impaired immunity and wound healing.

Xerostomia has been reported as one of the common oral symptoms in CKD patients (21). It occurs partly due to the medication or caused by the fluid intake restriction. Medications that cause xerostomia, such as diuretic and antihypertensive drugs, exert their effect through anticholinergic activity on the muscarinic acetylcholine receptor M_3_, or via centrally acting mechanisms on brain centre that reduce fluid secretion (22). Besides, xerostomia in patients with CKD can be due to the restriction of the fluid intake, which is necessary to accommodate the reduced excretory capacity of the kidney (23). On the other hand, it has been suggested that dry mouth sensation in patients with CKD is associated with bicarbonate decrease and calcium increase in the saliva, which can promote dental calculus formation (24). Uraemia associated with renal disease alter the inflammatory response to bacterial plaques in gingival tissue. This is because the uraemic state leads to the suppression of lymphocytic response, the dysfunction of granulocytes and the suppression of cell-mediated immunity. Consequently, uraemic patients are more susceptible to infection because of these changes (25).

In the present study, the mean of PPD in our subjects was 4.35 mm. Previous studies by Perozini et al. (3) and Gupta et al. (16) have reported lower PD mean values of 2.88 mm and 2.16 mm, respectively. Normal PD, which measures from pocket depth to GM, ranges from 1 mm–3 mm. PPD reading above 4 mm indicates increased PD, indicating that periodontitis is prevalent in our population compared to Brazil and India.

Deeper PDs are associated with higher levels of infiltrated connective tissues (causing more frequent bleeding, which is more difficult for patients and dentists to clean and maintain), higher levels of anaerobic pathogens, and a greater risk of disease progression and a frequent indication of loss of attachment (26).

As for CAL, our study showed increased attachment loss with 3.97 mm as compared to a study by Gupta et al. (16) which reported 1.77 mm. However, Perozini et al. (3) reported higher attachment loss of 4.30 mm in the pre-dialysis group in his study. Higher CAL indicates the extension of the destruction of periodontal support that has been destroyed around a tooth where there is a migration of epithelial attachment along the root surface and resorption of bone. The concentration of pathogens in sub-gingival plaque may reach a critical level required for initiation or progression of tissue destruction. Bacterial by-products gain access to the cellular constituents of the gingival tissues and activate cellular processes that are destructive to collagenous connective tissue (27). It can also be justified that poor oral hygiene and its cumulative effect over the years might have caused increased periodontal attachment loss in these patients. Frequent evaluation of the oral cavity in a longitudinal study, right from the pre-dialysis state, can give more meaningful and conclusive results.

CAL and PD measurements provide different information about the epidemiology of periodontitis. CAL is an indicator of cumulative tissue destruction, including past periodontal disease, while PD is an indicator of current disease status (14). This suggests that CKD patients in Perozini et al.’s study might have a past history of periodontal disease. This was, however, contradicted in Gupta et al.’s study, where there was no evidence of past periodontal disease among the CKD population.

Gupta et al.’s (16) study revealed that the mean of the periodontal parameters (PD and CAL) of haemodialysis patients were higher and severe compared to the pre-dialysis and control group. This finding is supported by Naugle et al.’s (28) study, which suggested that individuals on dialysis do not receive adequate periodontal care and the disease progresses unchecked. Self-reported information revealed poor attitude, as well as lack of awareness towards oral health care and its importance, seemed to be a trivial matter compared to the systemic illness that they cause. CKD patients seek dental treatment only on an emergency basis. As the dialysis itself is time-consuming, a time factor has to be considered to get dental treatment (28).

Moreover, the plaque index indicated an overall fair oral hygiene of pre-dialysis patients. This is similar to the mean of plaque index observed in pre-dialysis patients in Brazil, which showed fair oral hygiene (3). This is because patients with CKD consume antidiuretic drugs, which may impair salivary flow, thus reducing lubrication in the oral cavity and further promoting plaque accumulation. Apart from that, their oral health care practice was insufficient, which may worsen the oral hygiene condition (29).

In the present study, there was no correlation between periodontal parameters (PPD, CAL, plaque index and eGFR). Tawfig et al. (17) reported a similar result where there was no correlation between CAL and CKD staging; however, a correlation existed between the attachment loss and alkaline phosphatase enzymes. This non-correlation finding might be due to the study’s small sample size and probably because the focus was on one target group only (i.e., pre-dialysis patients). On the contrary, a study done among the general Korean population showed a correlation between periodontitis and decreased eGFR (30). The authors suggested that periodontitis has an indirect effect on decreased eGFR via hypertension and diabetes mellitus, in addition to its direct effect. Thus, a multicentre study could be conducted with a larger sample size that targets participants with decreased eGFR or other comorbidities to confirm this finding.

The findings of the present study are limited by a combination of confounding factors such as diet, smoking status, medications, improper oral health care and awareness regarding oral hygiene, which were not controlled. Moreover, our findings may be limited because only physical measurement of periodontal disease was made and not the effect of biochemical markers (serum creatinine, calcium and phosphorus) and inflammatory markers (interleukins) on periodontitis. There may be an important difference in the host response to bacterial challenges that need to be elucidated. Besides, this study was conducted in a single centre and was done within the stipulated time period, which led to the recruitment of only a few CKD patients. However, despite these limitations, we believe that our results are of interest because this is the first study of its kind in Malaysia.

## Conclusion

This study highlighted a greater prevalence and severity of periodontitis among pre-dialysis patients in a northeast Peninsular Malaysia tertiary hospital. CKD may be a risk factor for the periodontal disease; thus, it is important to improve patient oral health care practice and monitor their oral health condition. Dental and periodontal interventions should be done at the early stage of CKD, as there are possibilities of slowing down the progression of renal disease by reducing systemic inflammatory burden. Future studies with a multicentre approach and larger sample size are warranted to explore the magnitude of this problem.

## Figures and Tables

**Table 1:** Sociodemographic characteristics of patients (*n* = 46)

Variables	Mean (SD)	Frequency (%)(*n* = 46)
Age (years)	58.5 (12.8)	
Sex		
Male		37 (80.4)
Female		9 (19.6)
Ethnic		
Malay		44 (95.7)
Non-Malay		2 (4.3)
Comorbidities		
Hypertension		41 (89.1)
Diabetes mellitus		28 (60.9)
Cardiovascular disease		14 (30.4)
Hyperlipidemia		11 (23.9)
Gout		9 (19.6)
Benign prostate hyperplasia		6 (13.0)
CKD stage		
Stage III		19 (41.3)
Stage IV		27 (58.7)
eGFR (mL/min/1.73m^2^)	30.54 (11.76)	

**Table 2:** Mean of periodontal parameter in pre-dialysis patients

Periodontal parameter	Mean (SD)		

	Total of all cases of periodontitis	Mild periodontitis	Moderate-to-severe periodontitis
PPD (mm)	4.35 (0.99)	4.26 (0.26)	5.24 (0.36)
CAL (mm)	3.97 (0.97)	2.66 (0.62)	4.98 (0.73)
Plaque index (%)		47.9 (21.9)	

**Table 3:** Correlation between periodontal parameter and estimated glomerular filtration rate in pre-dialysis patients

	PPD		CAL		Plaque index	
	*r-*value	*P-*value	*r*-value	*P*-value	*r*-value	*P*-value
eGFR	−0.160	0.914	−0.135	0.372	0.005	0.974
